# Ventral Primary Hernia with Liver Content

**DOI:** 10.1155/2021/6698361

**Published:** 2021-05-31

**Authors:** Inès Dufour, Lancelot Marique, Thomas Valembois, Arnaud Ghilain, Gabriela Beniuga, Nicolas Tinton, Sabrina Urso, Benoît Colinet

**Affiliations:** ^1^Department of Pneumology, Grand Hôpital de Charleroi, Gilly, Belgium; ^2^Department of Abdominal and Transplantation Surgery, Cliniques Universitaires Saint-Luc, Bruxelles, Belgium; ^3^Department of Surgery, Grand Hôpital de Charleroi, Gilly, Belgium; ^4^Institute of Pathology and Genetics, Charleroi, Belgium

## Abstract

**Background:**

Herniation of the liver through the anterior abdominal wall is an extremely rare phenomenon. Most cases occur within an incisional hernia (mostly upper abdomen surgery or cardiac surgery). Only two reports mentioned liver herniation without previous abdominal incision. *Case Presentation*. We report the case of a 70-year-old woman presenting an epigastric swelling. Radiological findings showed a liver herniation in a primary ventral hernia. This case is the first to have been described requiring semiurgent hernia repair associated with partial liver resection.

**Conclusion:**

This case is, to the best of our knowledge, the first case of primary ventral hernia with liver content necessitating wedge resection of the left liver lobe.

## 1. Background

Herniation of the liver through the anterior abdominal wall is an extremely rare phenomenon. Most cases of liver herniation are due to diaphragmatic hernias (either congenital among children, either traumatic at adulthood). In a minority of cases of the childhood, the congenital anomaly is an abdominal wall defect with omphalocele [1].

In adults, hepatic herniation through the anterior abdominal wall is a really rare entity. Most occurred within an incisional hernia secondary to supraumbilical laparotomy for supramesocolic surgery (open cholecystectomy, orthotopic liver transplantation, etc.) or abdominal extension of sternal incision (e.g., CABG) [2, 3]. Only two reports mentioned liver herniation without previous abdominal incision. The first case is reported by Adeonigbagbe et al. [4] in 2000, when a 56-year-old woman presented with herniation of the liver through the rectus muscle. Obesity and hepatomegaly related to NASH were thought to be favoring factors. In 2001, Strul et al. [5] have reported a case of hepatic herniation in a 65-year-old male patient who presented with herniation of the left hepatic lobe through the abdominal rectus sheath, promoted by previous blunt abdominal trauma. In both cases, the patients have been managed conservatively.

This case is the first to describe semi-urgent hernia repair associated with partial liver resection. Herein, we discuss diagnosis and surgical management of this rare case.

## 2. Case Presentation

We report the case of a 70-year-old woman who was brought at the emergency service for evaluation of a sudden epigastric pain and nausea. She denied any history of trauma or heavy lifting. Her past medical history included total hip replacement, caesarean section, and a femoropopliteal artery bypass. She had no previous abdominal surgery history. She smoked for over 55 pack years.

Clinical examination revealed an important unreductible epigastric swelling. Biologically, haemoglobin was normal (Hb 13.3 g/dL), WBC count reached 20800/mm^3^ with presence of a discrete inflammatory syndrome (CRP 47 mg/L), and hepatic cytolysis ranged twenty times the normal values (GOT 796 U/L, GPT 895 U/L, and LDH 671 U/L).

**Figure 1:**
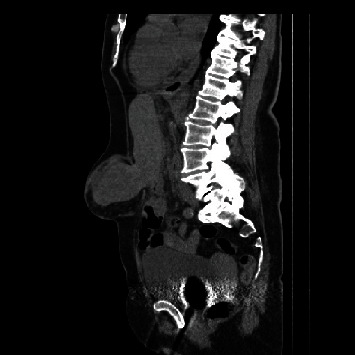
Sagittal section of noninjected CT abdominal scanner demonstrating herniation of liver segment III through the *linea alba*.

**Figure 2:**
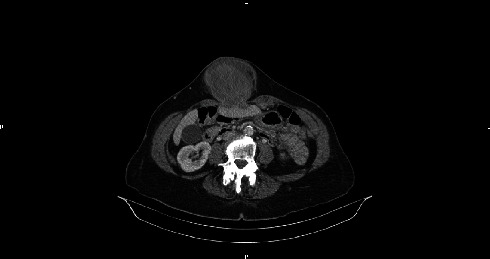
Axial section of injected CT abdominal scanner demonstrating herniation of liver segment III through the *linea alba*.

**Figure 3:**
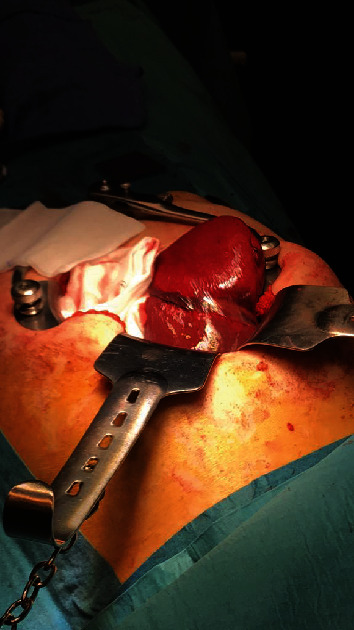
Preoperative view showing the hernia tightening ring on the left liver lobe.

**Figure 4:**
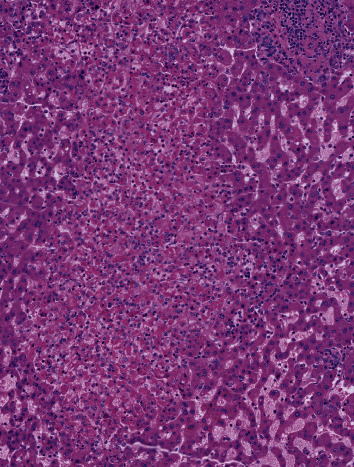
Inflammatory remodeling and microabscesses of the liver parenchyma.

An abdominal CT scanner showed huge epigastric hernia containing the left liver lobe associated with small amount of ascites within the hernia sac (Figures 1 and 2). A supra-umbilical laparotomy was performed for ventral hernia reparation. An important hernia sac was dissected and opened revealing epiploic and liver content. The exploration found a liver segment III that did not recover totally after liberation, and therefore, liver resection was performed (Figure 3). DynaMesh IPOM® hernia cure was performed in the retromuscular position. Liver anatomopathological analysis has shown subcapsular hemorrhagic suffusions associated with inflammatory remodeling and microabscesses (Figure 4).

Postoperative course was marked by an important ileus requiring a nasogastric tube for five days.

## 3. Discussion and Conclusions

In adults, liver herniation through the abdominal wall is a very scarce phenomenon. Most cases occurred within an incisional hernia (mostly upper abdomen surgery or cardiac surgery) [2, 3].

Two major questions arouse while performing the surgery: whether to resect the left liver lobe and whether to place or not a mesh.

Liver parenchyma is well vascularized by the hepatic artery and portal vein. Three elements in the case presentation were in favor of acute liver low perfusion of the incarcerated part: segment III hypodensity on injected CT scan, important inflammatory response with high WBC, and cytolysis edging twenty times the normal range.

Moreover, transient arterial ischemia can result in ischemic cholangiopathy [6]. Furthermore, bile duct ischemia is a well-described contributing factor for liver abscess secondary to pancreatoduodenectomy, liver transplantation, interventional techniques, or liver trauma [7]. Bile duct injury is very difficult to assess preoperatively and, even with a restored arterial blood flow, can occur at long term.

The rationale for performing a liver resection and for mesh placement in this case was put into balance with the contamination risk due to ischemic cholangiopathy or a liver abscess and with the recurrence risk with no mesh use.

It is now widely accepted that mesh repair for incarcerated or strangulated hernias is feasible with a great benefit of lower recurrence rates [8]. The use of mesh in clean-contaminated environment remains controversial. Some authors advocate avoiding the use of mesh in any level of contamination [9]. But a prospective 6-year study including 163 patients who underwent acutely incarcerated abdominal wall hernia mesh repair (48 required intestinal resection and anastomosis and 155 did not) showed no significant difference in terms of postoperative morbidities, wound infection, and recurrence rate [10]. The authors concluded that mesh hernia repair is crucial and is safe for repairing acutely incarcerated hernias, even in the case of intestinal resection.

For all these reasons, the ventral hernia cure using a mesh and partial left liver lobe resection were performed.

This case is, to the best of our knowledge, the first case of primary ventral hernia with liver content associated with wedge resection of the left liver lobe.

## Abbreviations

Hb: Haemoglobin
WBC: White blood cell
CRP: C-reactive protein
GOT: Glutamate oxaloacetate transaminase
GPT: Glutamic pyruvic transaminase
LDH: Lactate dehydrogenase
NASH: Nonalcoholic steatohepatitis
CABG: Coronary artery bypass graft.
